# Energy neutral: the human foot and ankle subsections combine to produce near zero net mechanical work during walking

**DOI:** 10.1038/s41598-017-15218-7

**Published:** 2017-11-13

**Authors:** Kota Z. Takahashi, Kate Worster, Dustin A. Bruening

**Affiliations:** 1 Department of Biomechanics, University of Nebraska at Omaha, Omaha, 68182 NE USA; 2 Medtronic Neurosurgery, Louisville, CO 80027 USA; 3 Department of Exercise Sciences, Brigham Young University, Provo, UT 84602 USA

## Abstract

The human foot and ankle system is equipped with structures that can produce mechanical work through elastic (e.g., Achilles tendon, plantar fascia) or viscoelastic (e.g., heel pad) mechanisms, or by active muscle contractions. Yet, quantifying the work distribution among various subsections of the foot and ankle can be difficult, in large part due to a lack of objective methods for partitioning the forces acting underneath the stance foot. In this study, we deconstructed the mechanical work production during barefoot walking in a segment-by-segment manner (hallux, forefoot, hindfoot, and shank). This was accomplished by isolating the forces acting within each foot segment through controlling the placement of the participants’ foot as it contacted a ground-mounted force platform. Combined with an analysis that incorporated non-rigid mechanics, we quantified the total work production distal to each of the four isolated segments. We found that various subsections within the foot and ankle showed disparate work distribution, particularly within structures distal to the hindfoot. When accounting for all sources of positive and negative work distal to the shank (i.e., ankle joint and all foot structures), these structures resembled an energy-neutral system that produced net mechanical work close to zero (−0.012 ± 0.054 J/kg).

## Introduction

Analyses of mechanical power, energy, and work are valuable for uncovering the role of the lower extremity during human locomotion, in particular, the foot and ankle structures. For example, studies in walking and running have shown that the ankle plantar flexor muscle-tendon structures are one of the major sources of positive work production (i.e., energy generation) within the lower limb^1–5^. These structures harness the elasticity of the Achilles tendon^6–9^ to supplement the body’s mechanical energy during push-off^5,10^. Recent students have also shown that the distal structures in the foot can show unique power and work distribution across various regions. Within the longitudinal arch, the surrounding muscle-tendon and fascia structures are thought to undergo stretch-shortening to absorb/store and generate/return energy during walking^11^ and running^11–13^. In the forefoot region, the metatarsophalangeal joints extend during push-off to yield negative work, and likely act to dissipate/absorb mechanical energy^14–16^. An increased understanding of the power and work contributions among structures of the foot and ankle will improve our knowledge of mechanisms underlying economical locomotion. Such insights could form a foundation for diagnosing pathologies affecting this anatomical region, and could inspire new designs of wearable device technology (e.g., prostheses, exoskeletons, and footwear).

One difficulty in partitioning power and work production within the foot and ankle is a lack of standard techniques for isolating the forces acting within each subsection of the foot. While multi-segment foot models can account for the motion (i.e., kinematics) of various subsections^17–19^, uncovering the kinetics (i.e., force, power, and work) among segments and joints is challenging. Traditional gait analysis techniques use a single force platform to estimate the total force acting underneath the foot^1,20^. Such approach, however, may not be suitable for isolating forces acting within the foot when more than one segments are in contact with the ground. For example, certain foot subsections could experience simultaneous opposing shear forces that may not be captured using a single force platform that measures only the net force acting on the foot^21,22^. Several investigators have used various approaches to partition the force and power/work profiles, including a phase-specific analysis when only a single foot segment (e.g., hallux) is in contact with the ground^15^, or combining a pressure mat with a force plate to partition the force into several components acting within the foot^16,23^. These techniques are useful in identifying joint kinetics using rigid-body mechanics, but could also miss contributions of power/work production due to structures that deform during ground contact.

**Figure 1:**
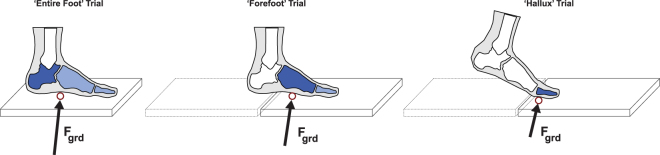
Participants walked in three different trials, where the foot made unique contact with ground-mounted force platforms: (1) Entire Foot Trial, (2) Forefoot Trial, and (3) Hallux Trial. The controlled foot placement trials enabled analyzing the forces acting on isolated regions within the foot. Using these force estimates, we quantified the total mechanical power and work contributions of various foot-ankle subsections by using a unified deformable (UD) segment analysis^23^. The UD analysis quantifies the summed contributions from all structures distal to a reference segment. By applying the analysis at four different segments (hallux, forefoot, hindfoot, and shank), this approach enabled partitioning of power/work within the foot and ankle.

The purpose of this study was to deconstruct the power and work profiles within the foot and ankle structures during human walking. The participants in this study were pediatric (ages 13.3 ± 3.1 years), but we feel that the results are generalizable for young adults since mature gait is reported to occur at around ages 7–8^24–26^. We used a controlled foot placement approach^27^ to isolate forces acting on a particular foot subsection (Fig. 1). Furthermore, we used an analysis that incorporates non-rigid mechanics^28–30^ to quantify power and work production within each subsection of the foot and ankle. This analysis, termed the unified deformable (UD) segment analysis, could offer several advantages over traditional rigid-body inverse dynamics approach. First, the UD analysis does not require defining of joint centers^29^, which could overcome difficulties in identifying articulations through surface anatomical landmarks, particularly in the mid-tarsal region^31^. Second, the UD analysis quantifies the total power and work production of all structures distal to a reference segment, as opposed to the total power/work production from tissue surrounding an isolated joint (i.e., joint power analysis). With the presence of various muscle-tendon and fascia structures that spans multiple joints within the foot (e.g., plantar fascia, foot intrinsic muscles), the UD analysis that combines the contributions of all of these structures may aid in the interpretation of the foot’s overall function. Therefore, we utilized the UD analysis in a segment-by-segment manner, and quantified the total power and work production within various regions of the foot and ankle during walking.

## Results

### Mechanical Power/Work from Structures Distal to Hallux

**Figure 2:**
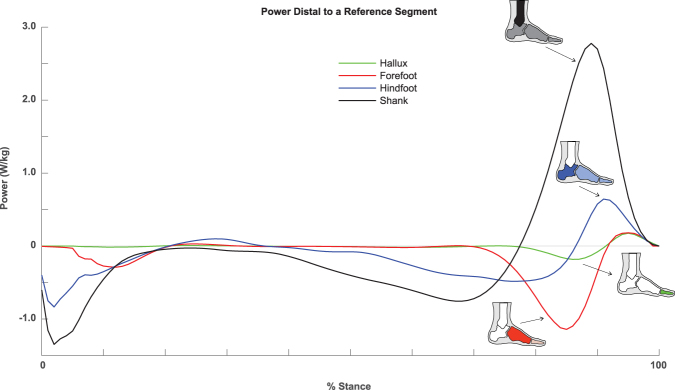
Stance period walking data of total mechanical power produced by structures distal to a reference segment: hallux (green), forefoot (red), hindfoot (blue), and shank (black). Estimates of distal to hallux and distal to forefoot powers were derived using force plate data from the Hallux Trial and the Forefoot Trial, respectively. Estimates of distal to hindfoot and distal to shank powers were derived using force plate data from the Entire Foot Trial.

**Figure 3:**
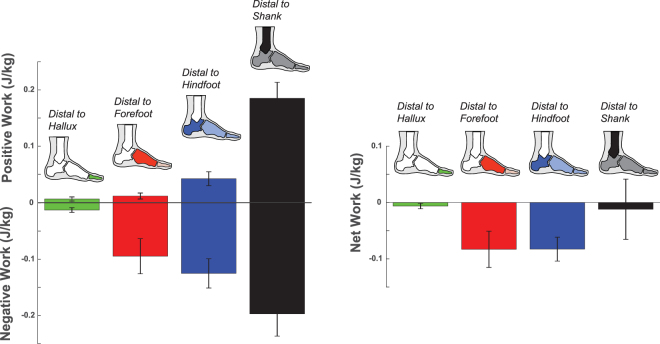
Positive, negative, and net work produced by structures distal to a reference segment: hallux (green), forefoot (red), hindfoot (blue), and shank (black). Both positive and negative work magnitudes generally increased from distal (hallux) to proximal (shank). The net work production distal to the shank is near zero (−0.012 ± 0.054 J/kg), suggesting that the foot-ankle complex is like an energy-neutral system.

Using the walking trial in which the participants’ entire hallux segment contacted a force platform (‘Hallux Trial’ – see Methods), we quantified the total mechanical power (Fig. 2) and work (Fig. 3) produced by the structures distal to the hallux. These structures exhibited very small production of negative and positive power and work. During the first ~90% of stance, these structures produced near zero mechanical power and work. In the last ~10% of stance, there was a small magnitude of negative power, followed by even smaller magnitude of positive power, accounting for negative work of −0.013 ± 0.004 J/kg and positive work of 0.006 ± 0.004 J/kg, amounting to net negative work of −0.006 ± 0.005 J/kg.

### Mechanical Power/Work from Structures Distal to Forefoot

Using the walking trial in which the participants’ forefoot and distal structures contacted a force platform (‘Forefoot Trial’ – see Methods), we quantified the power/work produced by the structures distal to the forefoot (i.e., soft tissue surrounding the metatarsal-phalangeal joints, hallux). These structures produced primarily negative power/work during stance. During the first ~70% of stance, these structures performed near zero mechanical power and work. Then, these structures performed largely negative power and work during the remainder of late stance. Overall, the structures distal to the forefoot performed −0.095 ± 0.031 J/kg of negative work, 0.012 ± 0.005 J/kg of positive work, amounting to net negative work of −0.083 ± 0.032 J/kg (Figs 2 and 3).

### Mechanical Power/Work from Structures Distal to Hindfoot

Using the walking trial in which the participants’ entire foot contacted a force platform (‘Entire Foot Trial’ – see Methods), we quantified the power/work produced by the structures distal to the hindfoot (i.e., soft tissue surrounding mid-tarsal joints, metatarsal-phalangeal joints, and hallux). These structures produced both negative and positive work during stance, and produced net negative work overall. Immediately after heel strike, there was a brief period of negative power and work. From ~40–90% of stance, there was another period of negative power and work. In the last ~10% of stance, these structures produced positive power and work prior to toe-off. Overall, the structures distal to the hindfoot performed −0.125 ± 0.026 J/kg of negative work, 0.042 ± 0.012 J/kg of positive work, amounting to net negative work of −0.083 ± 0.021 J/kg (Figs 2 and 3).

### Mechanical Power/Work from Structures Distal to Shank

From the ‘Entire Foot Trial’, we quantified the power/work produced by the structures distal to the shank (i.e., soft tissue surrounding the ankle joint, mid-tarsal joints, metatarsal-phalangeal joints, and hallux). These structures produced near equal magnitudes of negative and positive work during stance, resulting in near zero net work. During the first ~80% of stance, these structures performed negative power and work; characterized by an abrupt increase in negative power immediately following heel strike, and a gradual increase in negative power during ~30–80% of stance. During the last ~20% of stance, these structures performed positive power and work. Overall, the structures distal to the shank performed −0.197 ± 0.040 J/kg of negative work, 0.185 ± 0.028 J/kg of positive work, amounting to net work of −0.012 ± 0.054 J/kg (Figs 2 and 3).

## Discussion

The overall goal of this study was to quantify and partition the mechanical power and work production within isolated regions of the foot and ankle complex. Moreover, we conducted walking trials with controlled foot placements and used an analysis that incorporated non-rigid mechanics. Such approach identified disparate work profiles within various subsections. The three segmented foot model (hallux, forefoot, hindfoot) was particularly informative in describing unique energy profiles within the foot subsections. Most distally, the structures distal to the hallux exhibited near zero energetic activity during the majority of stance, and very small negative and positive work during push-off, contributing to net negative work of less than −0.01 J/kg. Distal to the forefoot, there was primarily negative work during push-off (net negative work of −0.083 J/kg). Such negative work could arise from the extension mobility of the metatarsal-phalangeal joints^14,16,27^ or from the compressibility of the fat pad underneath the metatarsal heads during push-off^32^. Our findings corroborate the inference that the structures distal to the forefoot overall dissipate or absorb energy and do not generate substantial energy during walking.

In structures distal to the hindfoot, elastic structures like the plantar fascia are thought to play a key role in positive work production during push-off^33^. For example, a recent *in-vivo* study of human running estimated that the plantar fascia could supply up to 3.1 Joules (or roughly 0.05 J/kg) of positive work through elastic energy return^13^. Our estimate of distal to hindfoot positive work during walking was similar in magnitude (0.04 J/kg), though our estimate also include summed contributions from other structures besides the plantar fascia (e.g., intrinsic and extrinsic foot muscles). Despite this positive work generated during push-off, the structures distal to the hindfoot performed net negative work of −0.08 J/kg during stance. This magnitude is comparable to the amount of net negative work done by the muscle-tendon structures surrounding the knee joint (−7.15 Joules or roughly −0.10 J/kg) during each stride of walking^3^. It is currently unclear where the foot’s energy goes, specifically how this negative work is being used by the body. It is possible that a portion of this negative work could be transferred proximally to the shank through multi-articulating muscles^34^ or a portion of the negative work could be dissipated as heat^35,36^.

**Figure 4:**
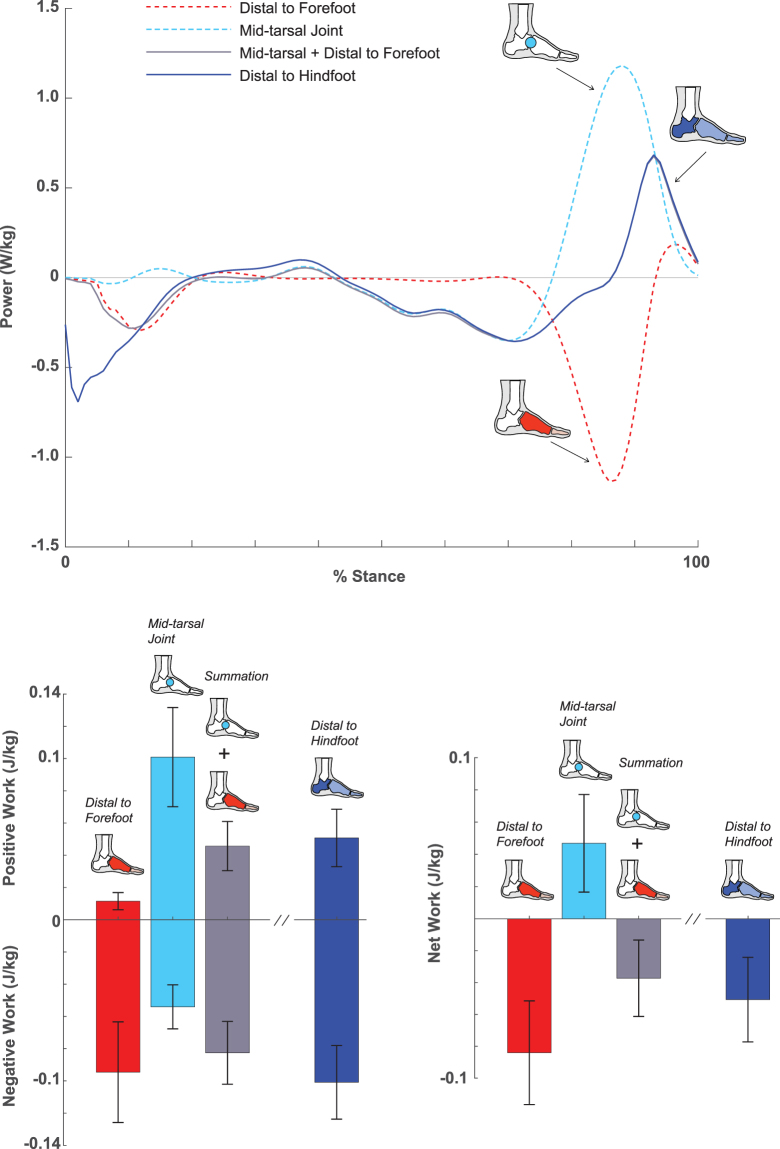
Stance phase data (from the Forefoot Trial) of mechanical power and work production within subareas of the foot: distal to forefoot (red), mid-tarsal joint (turquoise), summed midtarsal joint and distal to forefoot (gray), and distal to hindfoot (blue). The mid-tarsal joint generated substantial positive power and work during push-off (last ~20% of sdtance), while at the same time, the structures distal to the forefoot performed negative power and work. Overall, all structures distal to the hindfoot produced net negative work.

To further understand the mechanical output distal to the hindfoot, we performed a secondary analysis from the Forefoot Trials (Fig. 4). This involved partitioning the contributions of the mid-tarsal joints using a joint power based technique as well as the structures distal to the forefoot (See Methods). In agreement with prior studies^12,16,27^, the mid-tarsal joints produced largely positive power and work, contributing to net positive work of 0.047 J/kg during the entire period of stance. Yet, the positive work production from the mid-tarsal joints during push-off were largely offset by the simultaneous negative work by structures distal to the forefoot (e.g., the metatarsal-phalangeal joints)^12,13^. Such opposing work production from the mid-tarsal joints and distal structures may be primarily a consequence of engaging the Windlass mechanism^37^. This mechanism comes into play when the metatarsal-phalangeal joints extend during late stance, which effectively lengthens the plantar fascia (and possibly other muscle-tendon structures that comprise the longitudinal arch) that could induce tension and facilitate positive work production at the mid-tarsal joints during push-off^13,38^. Because fascia and most muscle-tendon structures cross both the metatarsal-phalangeal and mid-tarsal joints, it is likely that the structures responsible for producing positive work at the mid-tarsal joints are the same structures that produce negative work at the metarsal-phalangeal joints. Thus, rather than separating the two components (such as through joint power based calculations), our analysis combines the contribution of all distal structures relative to the hindfoot and thus may aid in the interpretation of the overall function of the human foot.

Another important feature of distal to hindfoot structures is the energy absorption immediately after heel strike (first ~10% of stance). This negative power is absent in the mid-tarsal joint (Fig. 4), and thus is likely associated with the forces acting beneath the hindfoot segment (i.e., heel pad) that induces deformation. The human heel pad is known to have viscoelastic behavior that shows substantial hysteresis when force is applied^39,40^, which could allow the heel to function as a shock absorber following initial contact during walking^41^. This dissipative capacity of the heel region thus appears to contribute to the overall net negative work of the foot during the entire stance period.

Proximal to the hindfoot, the ankle joint is one of the body’s major sources of positive work during walking^1–5^. Yet, when combining all contributions of structures distal to the shank (including the ankle joint and foot structures), the overall output strikingly resembled an energy neutral system that produced near zero net work (or slightly net negative). Moreover, the muscle-tendon structures of the ankle joint perform net positive work^1–3,30,42^ while the foot structures perform net negative work^30,43^ (also see Supplementary Figure 1). These results are consistent with a recent finding showing the ankle-foot system produces near net zero work across a range of walking speeds; in particular, Takahashi *et al*. found net negative work of −0.04 J/kg while walking at ~1.38 m/s^30^, whereas our study found net negative work of −0.01 J/kg while walking at ~1.33 m/s.

In theory, walking at a steady-state speed on level ground requires zero net mechanical work from the entire body^44–46^, since the positive and negative work required to accelerate/decelerate the body or the work needed to raise/lower the center of mass must be balanced. It appears that the zero net work behavior may also be applicable to the foot and ankle complex overall. While these distal limb structures contain a mixture of structures that are elastic (e.g., Achilles tendon, plantar fascia) or viscoelastic (e.g., plantar fat pad), as well as muscles that can produce work actively (e.g., ankle plantar flexor, intrinsic and extrinsic foot muscles), the overall magnitudes of positive and negative work are largely balanced. This energy neutral behavior could be a hallmark of a healthy foot and ankle system, in which these distal limb structures coordinate the overall work production. Our segment-by-segment analysis could open new avenues to investigate how certain pathologies may disrupt the overall mechanical work distribution within this anatomical region, or it could inform how wearable devices (e.g., prostheses, exoskeletons, orthoses, footwear) could be optimized.

Mechanics of an energy neutral foot-ankle system may be emulated by devices that do not necessarily require battery-operated motors and actuators. While the recent emergence of powered devices such as exoskeletons^47–51^ and prostheses^52–54^ have shown great promise for improving mobility, more simplistic unpowered devices have also shown great capacity for improving locomotion outcomes. For example, using an unpowered ankle exoskeleton composed of a spring and a clutch, healthy humans can reduce whole-body metabolic energy cost by as much as 7%^55^. Furthermore, recent studies showed that physically-fit amputees who use unpowered ankle-foot prostheses on one limb can exhibit metabolic energy cost of walking comparable to able-bodied counterparts^56,57^. Our comprehensive examination of foot and ankle structures may facilitate fine-tuning of unpowered device designs, such as modifications in stiffness^58–61^, damping^62^, and geometry^63,64^ that could closely replicate the overall work production of the biological foot and ankle.

The present study has a few limitations. First, isolating the kinetics of foot subsections required a controlled foot placement on a force platform (such as during the Hallux Trial or the Forefoot Trial). These trials could potentially alter the participants’ walking patterns, and require data from the outer edge of a force platform, which may be more prone to inaccuracy. We therefore performed a secondary analysis to compare the total power/work produced by structures distal to the hindfoot and the shank across the different walking trials (Supplementary Figure 2 and Supplementary Table 1). While there were some statistically significant differences in work production, the overall behavior regarding the net work production from hindfoot and shank distal structures were preserved. That is, regardless of the walking trial, distal to hindfoot structures produced net negative work during stance and distal to shank structures produced near zero net work (Supplementary Figure 2 and Supplementary Table 1). Second, the subjects recruited for this study were pediatric (ages 13.3 ± 3.1 years), and thus our findings may not necessarily be generalizable for other populations. However, gait is considered mature by ages 7–8^24–26^, and the mass-normalized power distal to the shank appears comparable to published studies in healthy adults^29^. Lastly, our estimates of mechanical power/work derived from this study cannot parse out the contributions from passive structures (such as heel pad, Achilles tendon, plantar fascia) versus active muscle contractions, nor can it identify how mechanical energy is transferred between segments via multi-articulating muscles. Further analyses integrating *in-vivo* imaging techniques, such as ultrasound imaging^65–67^, as well as musculoskeletal modeling^10,13,34^ may further shed insights on the exact source of mechanical work within the foot and ankle.

## Conclusions

Through a segment-by-segment analysis, we partitioned the mechanical work production of the human foot and ankle during walking. Our results revealed disparate energetic profiles within various subsections of the foot and ankle. The magnitude of positive work generation increased systematically from most distal segment (hallux) to proximal (shank). The overall work output of the foot and ankle resembled an energy-neutral system, in that the net work is near zero (or slightly net negative). These results revise our fundamental understanding of foot and ankle functions during locomotion, and could inspire novel approaches for wearable device technology, such as prostheses, exoskeletons, and footwear.

## Methods

### Subject Profiles

We re-analyzed the data collected in a previous study^27,68^. Data from 14 participants (5 females and 9 males, ages 13.3 ± 3.1 years, height 1.57 ± 0.17 m, body mass 51.4 ± 17.2 kg; means ± standard deviation) were included for this study. All participants signed an informed consent to participate in this study. The study was approved by the Institutional Review Board (IRB) from Hamot Hospital (Erie, PA, USA) and the University of Delaware (Newark, DE, USA). The methods were performed in accordance with the IRB-approved protocol.

### Multi-Segment Foot Model

The details of the skin-based marker set for the multi-segment foot model were described in a previous study^27,68^. Briefly, the model included a shank segment, and three segments within the foot: hindfoot, forefoot, and hallux. The hallux and forefoot segments were separated by the first metatarsal-phalangeal joint, defined by projecting the dorsal 1^st^ metatarsal head marker downward half the distance to the floor. The forefoot and the hindfoot segments were separated by the mid-tarsal joints, defined by the midpoint between the markers placed on the lateral centroid of the cuboid bone and the medial prominence of the navicular bone. The hindfoot and shank segments were separated by the ankle joint, defined by the midpoint between the markers placed on the medial and lateral malleoli, with an additional correction that accounts for combined talocrural and subtalar joint motion^69^.

### Controlled Foot Placement for Isolating Kinetics of the Foot and Ankle

The participants walked at a self-selected speed along a floor containing two adjacent force platforms (AMTI, Watertown MA, USA), that were separated by approximately 2 mm. The force platform data was sampled at 1560 Hz. A 10-camera motion capture system (Vicon, Oxford England, UK) was used to collect motion of the skin-based markers (sampled at 120 Hz). Motion capture data and force platform data were low-pass filtered at 6 Hz and 100 Hz, respectively. The participants performed three different trial types. First, the participants walked across the floor, where trials were deemed successful if the entire foot contacted a single force platform (i.e., ‘Entire Foot Trial’). Second, the participants walked with a controlled three-step approach; the third step was intended to have the entire hallux segment contact the anterior force platform, while all structures proximal to the metatarsal-phalangeal joints contact the posterior force platform (i.e., trial to isolate kinetics of the hallux: ‘Hallux Trial’). Third, the participants walked with a controlled three-step approach; the third step was intended to have the forefoot and hallux contact the anterior force platform, while all structures proximal to the mid-tarsal joints contact the posterior force platform (i.e., trial to isolate the kinetics of forefoot and distal structures: ‘Forefoot Trial’).

It is important to note that the participants were not instructed to target their foot placement to achieve the desired contact with the force platforms. Instead, the participants were instructed to walk as normal as possible, while the investigators adjusted the starting positions to ensure appropriate foot contact. Obtaining proper foot contact with the force platforms typically required 15–30 trials, and proper foot contacts were verified visually by two video cameras located on either sides of the force platforms^27^. The self-selected speeds for the Entire Foot Trial, the Forefoot Trial, and the Hallux Trial were 1.33 ± 0.12 m/s, 1.28 ± 0.15 m/s, and 1.28 ± 0.12 m/s, respectively.

### Mechanical Power and Work Analyses

To quantify mechanical power within various subsections of the foot-ankle complex, we used a unified deformable (UD) segment analysis^29,70^. The UD segment model is a hybrid segment composed of a rigid component (i.e., reference segment) and a distal deformable component. The UD analysis quantifies the total power due to all structures distal to a chosen reference segment. For example, when the UD analysis is applied at the shank segment, it captures the summed contribution of the ankle joint and all foot structures. When the UD analysis is applied at the hindfoot segment, it captures the summed contribution of the mid-tarsal joint, metatarsal-phalangeal joint, hallux segment, and deformation of soft tissue surrounding this anatomical region. In this study, we quantified four separate UD-based power profiles (${P}_{U{D}_{ref}}$) derived from four different reference segments: hallux, forefoot, hindfoot, and shank. For each reference segment, we applied the following computation:

1 $${P}_{U{D}_{ref}}={\bar{F}}_{GRF}\cdot {\bar{v}}_{CO{P}_{ref}}+{\bar{M}}_{free}\cdot {\bar{\omega }}_{ref}$$

${P}_{U{D}_{ref}}\,$is the total mechanical power of structures distal to a chosen reference segment (shank, hindfoot, forefoot, or hallux). ${\bar{F}}_{GRF}$ and ${\bar{M}}_{free}$ are the ground reaction force and free moment, respectively. ${\bar{\omega }}_{ref}$ is the angular velocity of the reference segment (based on a rigid body assumption). ${\bar{v}}_{CO{P}_{ref}}\,$is the translational velocity of a point on the reference segment that is coincident with location of the center-of-pressure. ${\bar{v}}_{CO{P}_{ref}}$ is computed as:

2 $${\bar{v}}_{CO{P}_{ref}}={\bar{v}}_{CO{M}_{ref}}+({\bar{\omega }}_{ref}\,\times \,{\bar{r}}_{CO{P}_{ref}})$$

${\bar{v}}_{CO{M}_{ref}}$ is the translational velocity of the center-of-mass of the reference segment (based on a rigid body assumption). ${\bar{r}}_{CO{P}_{ref}}$ is the displacement of the center-of-pressure, relative to the center-of-mass of the reference segment.

To compute ${P}_{U{D}_{ref}}$ at the hallux, we used the force platform data (${\bar{F}}_{GRF}$, ${\bar{M}}_{free}$, and center-of-pressure) from the Hallux Trial (Fig. 1). To compute ${P}_{U{D}_{ref}}$ at the forefoot, we used the force platform data from the Forefoot Trial. To compute ${P}_{U{D}_{ref}}\,$at the hindfoot and at the shank, we used the force platform data from the Entire Foot Trial. By performing separate UD analysis at four different segments (shank, hindfoot, forefoot, and hallux), this approach enabled partitioning of power/work contributions of ankle and foot structures in a segment-by-segment manner. The mechanical work profiles were quantified from the time-integral of ${P}_{U{D}_{ref}}$ during the stance period.

To partition the mechanical power and work contributions from the structures distal to the hindfoot, we performed a secondary analysis from the ‘Forefoot Trial’ that included foot contact with two force platforms (Fig. 4). First, by combining estimates from both the anterior and posterior force plates, we computed a single ${\bar{F}}_{GRF}$, ${\bar{M}}_{free}$, and center-of-pressure data^21,27^. Using the combined force plate measures, we computed ${P}_{U{D}_{ref}}\,$at the hindfoot (using methods described previously). Then, using data from only the anterior plate, we quantified ${P}_{U{D}_{ref}}\,$at the forefoot (as described above) as well as mid-tarsal joint power. The mid-tarsal joint power was quantified using a 6 degree-of-freedom joint power method that incorporates both rotational and translational components of movement^71,72^. Then the summation of mid-tarsal joint and ${P}_{U{D}_{ref}}\,$at the forefoot was taken. This secondary analysis from the ‘Forefoot Trial’ allowed us to a gain deeper knowledge of the mechanical sources of power from the structures distal to the hindfoot (i.e., how much power/work could come from the mid-tarsal joint and structures distal to the forefoot).

## Electronic supplementary material

Supplementary Information
