# Supplementary material for: Energy neutral: the human foot and ankle subsections combine to produce near zero net mechanical work during walking
Source: Sci Rep. 2017 Nov 13;7:15404. doi: 10.1038/s41598-017-15218-7 (PMC5684348; doi:10.1038/s41598-017-15218-7)
Supplement: Supplementary file 1 — Supplementary Information [file 41598_2017_15218_MOESM1_ESM.pdf]

Supplementary Information

Energy neutral: the human foot and ankle subsections combine to produce near zero net mechanical work during walking

***Kota Z. Takahashi[1]**

Kate Worster[2]

Dustin A. Bruening[3]

corresponding author

[1] Department of Biomechanics, University of Nebraska at Omaha

Email: ktakahashi@unomaha.edu

Phone: 1-402-554-4184

[2]Medtronics Neurosurgery, Louisville, CO

Email: worsterk@gmail.com

Phone: 1-303-809-5242

[3] Department of Exercise Sciences, Brigham Young University

Email: dabruening@byu.edu

Phone: 1-801-422-1420

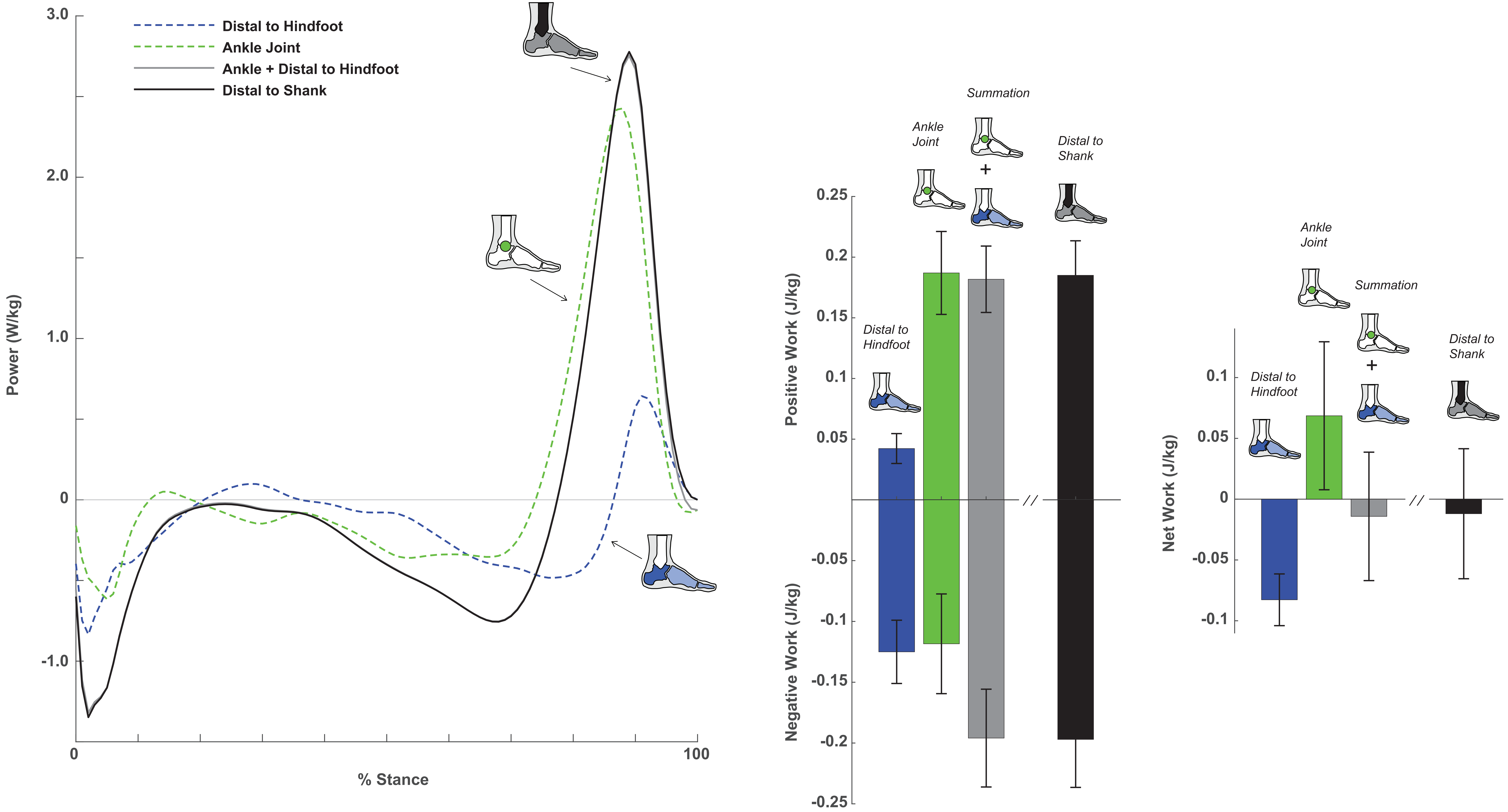

Supplementary Figure S1: Stance phase data (from the Entire Foot Trial) of mechanical power and work contributions from subareas within the foot/ankle system: distal to hindfoot (blue), ankle joint (green), summed ankle joint and distal to hindfoot (gray), and distal to shank (black). The ankle joint produced net positive work, while distal to hindfoot structures produced net negative work. The combined effect yielded near zero net work from structures distal to the shank. The distal to shank power (black) estimates agreed well with the summed ankle and distal to hindfoot power (gray) throughout the entire stance period.

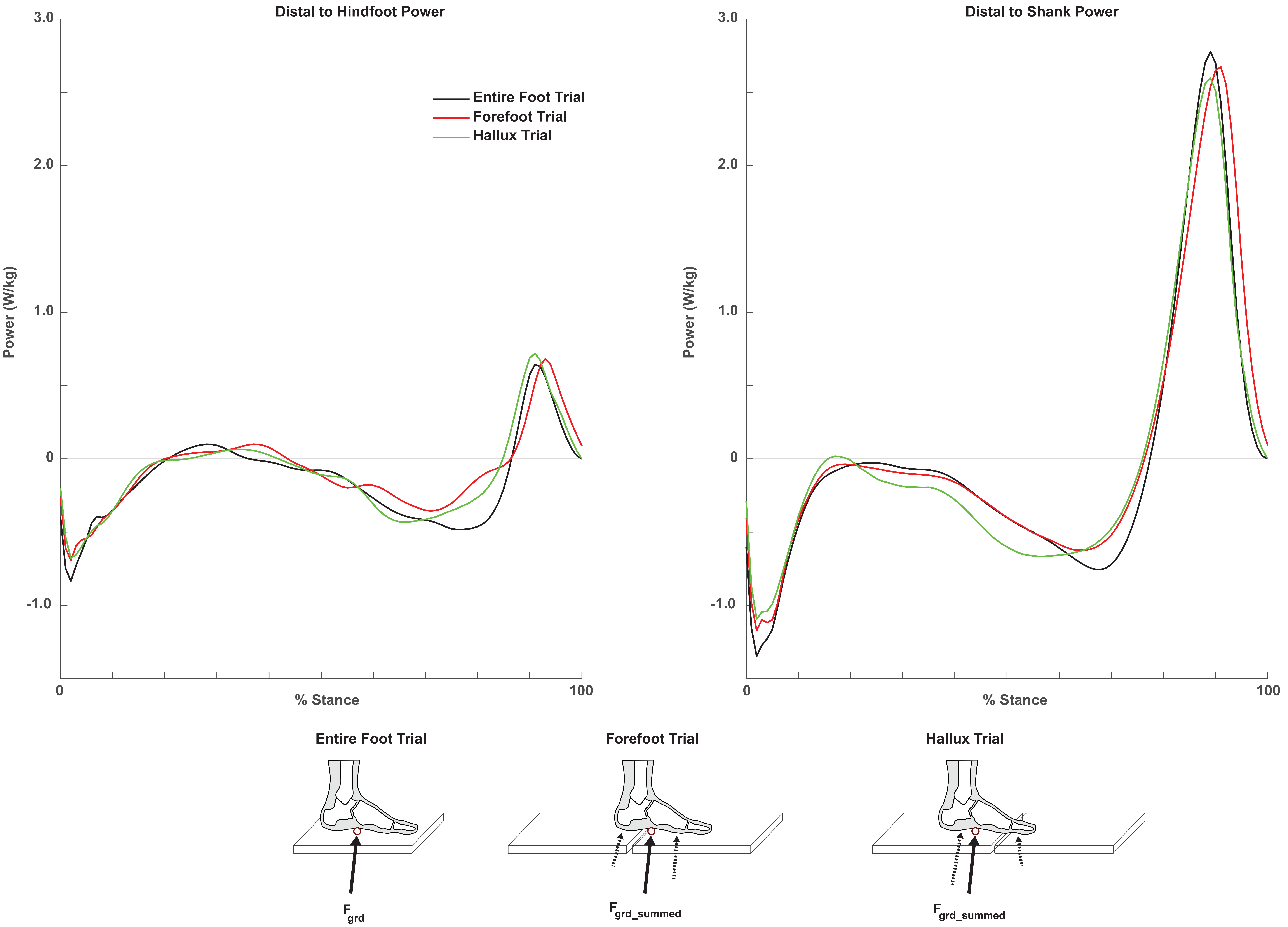

Supplementary Figure S2: Comparison of distal to hindfoot power and distal to shank power from three separate walking trials: Entire Foot Trial (black), Forefoot Trial (red), and Hallux Trial (green). In the Forefoot Trial and the Hallux Trial, data from the two force plates were summed to create a single ground reaction force vector. The combined force estimates were used to quantify the total power from structures distal to the hindfoot and distal to the shank.

	Entire Foot Trial	Forefoot Trial	Hallux Trial
**Distal to Hindfoot Work**			
Pos Work (J/kg)	0.042 ± 0.012	0.051 ± 0.018<br>(p = 0.065)	0.052 ± 0.015<br>*(p = 0.035)
Neg Work (J/kg)	-0.125 ± 0.026	-0.101 ± 0.023<br>*(p = 0.001)	-0.116 ± 0.022<br>(p = 0.2678)
Net Work (J/kg)	-0.083 ± 0.021	-0.050 ± 0.023<br>*(p < 0.001)	-0.064 ± 0.022<br>*(p = 0.0265)
**Distal to Shank Work**			
Pos Work (J/kg)	0.185 ± 0.028	0.205 ± 0.047<br>*(p = 0.0417)	0.193 ± 0.047<br>(p = 0.6074)
Neg Work (J/kg)	-0.197 ± 0.039	-0.185 ± 0.048<br>(p = 0.1354)	-0.204 ± 0.058<br>(p = 0.3932)
Net Work (J/kg)	-0.012 ± 0.053	0.020 ± 0.069<br>*(p = 0.0157)	-0.011 ± 0.068<br>(p = 0.9452)
**Walking Speed (m/s)**	1.33 ± 0.12	1.28 ± 0.16<br>(p = 0.1846)	1.28 ± 0.12<br>(p = 0.1049)

**Root Mean Square Difference (relative to Entire Foot Trial)**	Forefoot Trial	Hallux Trial
Distal to Hindfoot Power (W/kg)	0.1221	0.091
Distal to Shank Power (W/kg)	0.1973	0.1495

Supplementary Table S1: Compared to the Entire Foot Trial, the Forefoot Trial and the Hallux Trial produced some differences in mechanical work output distal to the hindfoot and distal to the shank. P-values represented paired t-test relative to the Entire Foot Trial, and asterisks were used to denote significant difference at $\alpha = 0.05$. There were no significant differences in walking speed across the trials. The root mean square differences (relative to the Entire Foot Trial) of the mechanical power estimates during the entire stance period were less than 0.2 Watts/kg.
